# Echocardiography-guided percutaneous closure of perimembranous ventricular septal defects without arterial access and fluoroscopy

**DOI:** 10.1186/s12887-019-1687-0

**Published:** 2019-08-31

**Authors:** Haisong Bu, Yifeng Yang, Qin Wu, Wancun Jin, Tianli Zhao

**Affiliations:** 1 Department of Cardiovascular Surgery, The Second Xiangya Hospital, Central South University, 139 Renmin Road, Changsha, 410011 Hunan China; 2 Department of Echocardiography, The Second Xiangya Hospital, Central South University, 139 Renmin Road, Changsha, 410011 Hunan China

**Keywords:** Percutaneous device closure, Perimembranous ventricular septal defect, Transesophageal echocardiography, Femoral vein, Radiation prevention

## Abstract

**Background:**

Traditional percutaneous device closure of perimembranous ventricular septal defects (PmVSDs) is a minimally invasive technique, but can result in high radiation exposure and can result in potential arterial complications. Here, we aimed to assess the safety and feasibility of device closure of PmVSDs via the femoral vein approach under transesophageal echocardiography (TEE) guidance in children.

**Methods:**

From January 2014 to December 2017, a total of 46 PmVSD patients (mean age, 6.5 ± 2.3 years [range, 4.2–12.0 years]; mean body weight 22.1 ± 6.6 kg [range, 16.0–38.5 kg]; VSD diameter, 4.1 ± 0.6 mm [range, 3.2–5.0 mm]) underwent attempted transcatheter closure via the femoral vein approach under the guidance of TEE without fluoroscopy.

**Results:**

The transcatheter occlusion procedure under TEE guidance was successful in 44 (95.7%) patients. Surgery was necessary in 2 (4.3%) patients. The procedure duration was 28.2 ± 8.7 min (range, 12.0–42.0 min). One patient had immediate post-operative trivial residual shunt and three patients had immediate incomplete right bundle branch block (IRBBB) after operation; the new IRBBB in 1 case was noted in the first postoperative month. No residual shunt was noted at 3 months after the procedure, and no intervention related complications were detected at 1–24 months follow-up.

**Conclusions:**

Percutaneous device closure of PmVSDs under TEE guidance solely by femoral vein approach is effective and safe, avoids radiation exposure, potential arterial complications and a surgical incision.

**Electronic supplementary material:**

The online version of this article (10.1186/s12887-019-1687-0) contains supplementary material, which is available to authorized users.

## Background

Perimembranous ventricular septal defect (PmVSD) is one of the most common congenital cardiac malformations. Since the first successful on-pump PmVSD closure, surgical repair through median sternotomy under cardiopulmonary bypass (CPB) has been considered the gold standard in pediatric cardiac surgery. As catheterization techniques develop and spread, traditional transcatheter VSD closure has been used to date, but result in radiation exposure [1] and potential arterial complications [2, 3]. In an effort to reduce injury related to fluoroscopy and angiography, another alternative technique is direct ventricular puncture using to close the VSD with a beating heart using an occluder recently came into practice. This method is widely used in China [4–8] and other countries [9–12]. However, there are still some disadvantages with this technique, including surgical trauma, incision, and scarring. In this study, we established a new method for transcatheter VSD closure through femoral venous route under the guidance of TEE, without accessing the femoral artery or the employment of fluoroscopy. This single-centered study aims to access the feasibility and safety of this new strategy.

## Methods

### Patients

We enrolled 46 patients with isolated PmVSDs at Second Xiangya Hospital between January 1, 2014, and December 31, 2017 which included 26 male and 20 female patients (mean age, 6.5 years (range 4.2–12.0 years); mean body weight 22.1 ± 6.6 kg [range, 16.0–38.5 kg]; VSD diameter, 4.1 ± 0.6 mm [range, 3.2–5.0 mm]) (Table 1). The PmVSD were detected by pre-operation transthoracic echocardiography (TTE) (Vivid™ E9; GE Healthcare, Little Chalfont, United Kingdom) with color and Doppler interrogation from sub-xyphoid, apical, and parasternal views.

**Table 1:** Patient characteristics

Variable	Values
Total number (n)	46
Male/female (n)	26/20
Mean age (years)	6.5 ± 2.3 (range, 4.2–12.0)
Mean weight (kg)	22.1 ± 6.6 (range, 16.0–38.5)
PmVSD diameter (mm)	4.1 ± 0.6 (range, 3.2–5.0)
Other malformations	
IRBBB (n)	4
Aortic valve regurgitation (trivial) (n)	1
Mitral valve regurgitation (n)	0
Tricuspid valve regurgitation (mild) (n)	1
Outcome as expected (n)	44
Switch to open heart surgery (n)	2
Device size (mm)	6 mm,7 mm
Operative duration (min)	28.2 ± 8.7 (range, 12.0–42.0)
Mechanical ventilation duration (min)	65.2 ± 5.6 (range, 56.0–78.0)
Intensive care unit duration (h)	2.1 ± 0.1 (range, 2.0–2.4)
Total length of stay (day)	2.7 ± 0.2 (range, 2.5–3.0)
In-hospital complication	
IRBBB (n)	3
Residual shunt (n)	1 (width, 1 mm; flow rate < 2.0 m/s)
Follow-up complication (Medium)	
IRBBB (n)	1
Follow-up complication (Long-term)	
IRBBB (n)	0
Follow-up time (months)	21.8 ± 4.7 (range, 12–24)

Values are presented as mean ± standard deviation

*PmVSD* perimembranous ventricular septal defect, *IRBBB* incomplete right bundle branch block

The patient selection criteria were as follows: 1) a left-to-right shunt whose Qp/Qs was ≥1.5 due to isolated PmVSD; 2) patient age between 2.0 years and 13.0 years; 3) patient weight > 12.5 kg; 4) maximum PmVSD diameter of ≤6 mm and minimum PmVSD diameter of ≥3 mm; and 5) subaortic rim (distance from the defect to the aortic valve) of > 2 mm. Patients with the following conditions were excluded: 1) more than a mild degree of aortic valve prolapse or regurgitation; 2) malalignment PmVSD; and 3) coexisting cardiac anomalies requiring correction during the same procedure.

Informed consent was obtained from the parents of all participants, and the study was approved by the Committee on Clinical Applications at the Second Xiangya Hospital. All patients were followed up with clinical examination, TTE, and electrocardiography at 1, 3, and 6 months, and 1 and 2 years after the procedure.

### Device and delivery system

**Fig. 1:**
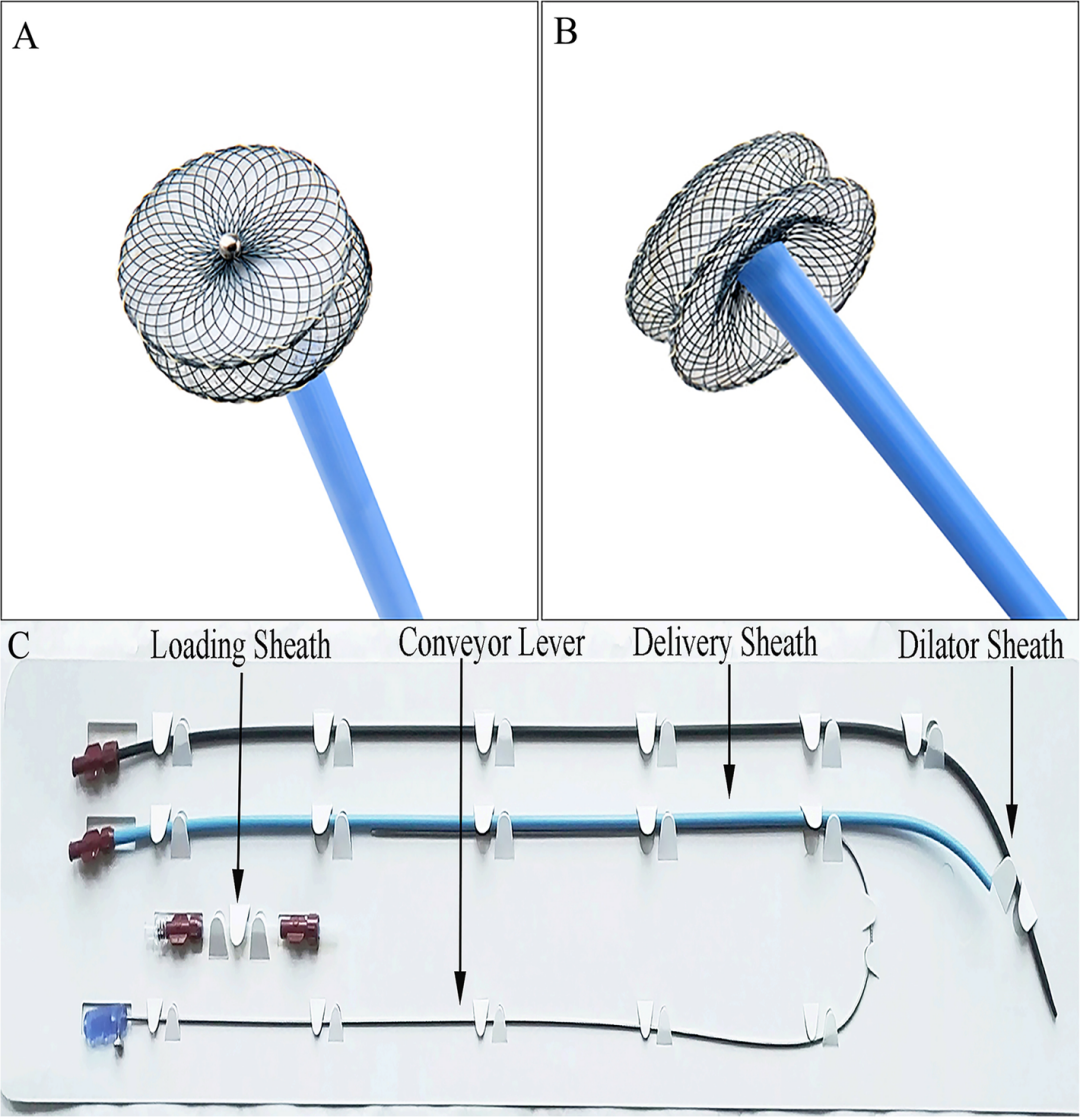
Double-disk S-pmVSO devices and Delivery systems. **a** Front view of an S-pmVSO; **b** lateral view of an S-pmVSO; **c** Delivery systems. S-pmVSO: symmetrical concentric PmVSD occluder

The VSD occluder (Fig. 1a and b; Shanghai Shape Memory Alloy Co. Ltd., Shanghai, China) was described in previous report [6]. In brief, this occluder is double-disk symmetrical concentric PmVSD occluder (S-pmVSO), and both discs were 2 mm larger than the waist. The waist was 4-14 mm long, with 3.5–4.5 mm diameter for its full length, corresponding to the size of the VSD in patients. The occluder size is selected according to the VSD diameter measured by TEE, with its diameter 2 mm larger than the VSD diameter.

The device is attached by a recessed microscrew to a delivery cable made of stainless steel (Fig. 1c). It is delivered through a 7F–8F long sheath. For introduction into the delivery sheath, the device is pulled into a special loader. The delivery system is also manufactured by Shanghai Shape Memory Alloy Co. Ltd., Shanghai, China.

### Procedure

The procedure was performed in a routine operating room under TEE guidance without fluoroscopy. All patients underwent general anesthesia with intubation in a supine position. TEE (Vivid E9 Dimension, bought from General Electric Company, Horten, Norway) was inserted into the middle esophagus. 9 T for patients less than 15 kg, 6 T for patients more than 15 kg. TEE studies were performed to evaluate the position, size, shape, direction of shunt flow, and the surrounding rims (particularly the subaortic rim) of the PmVSD prior to the procedure.

**Fig. 2:**
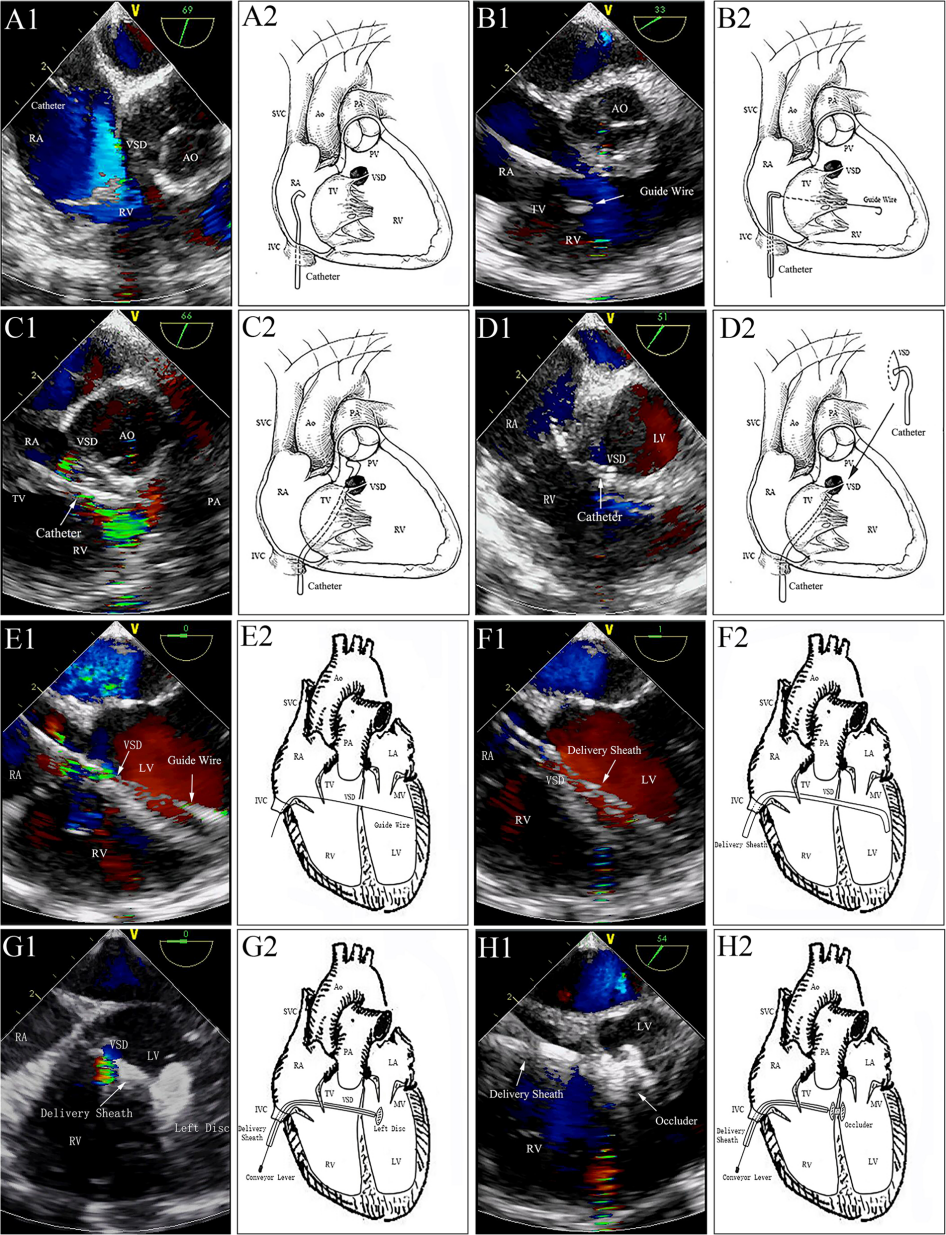
Delivery tract establishment and device deployment. A1&A2: The catheter was progressively withdrawn to reach the middle of the TV; B1&B2: The guidewire was delivered to the RV via the TV; C1&C2: The catheter was advanced into the right ventricular outflow tract; D1&D2: The tip of the catheter was entered into the LV under TEE guidance. E1&E2: Guidewire advanced through the defect and into the LV under TEE guidance. F1&F2: Sheath advanced to the LV tracked by TEE. G1&G2: Left disc of the S-pmVSO was deployed in the LV. H1&H2: Right side of the device was deployed by retracting the delivery sheath while applying slight tension on the cable. LV: left ventricle; LA: left atrium; RA: right atrium; RV: right ventricle; AO: aorta; VSD: ventricle septal defect; TV: tricuspid valve; PV: pulmonary artery valve; MV: mitral valve; PA: pulmonary artery; SVC: superior vena cava; IVC: inferior vena cava

**Figure 3:**
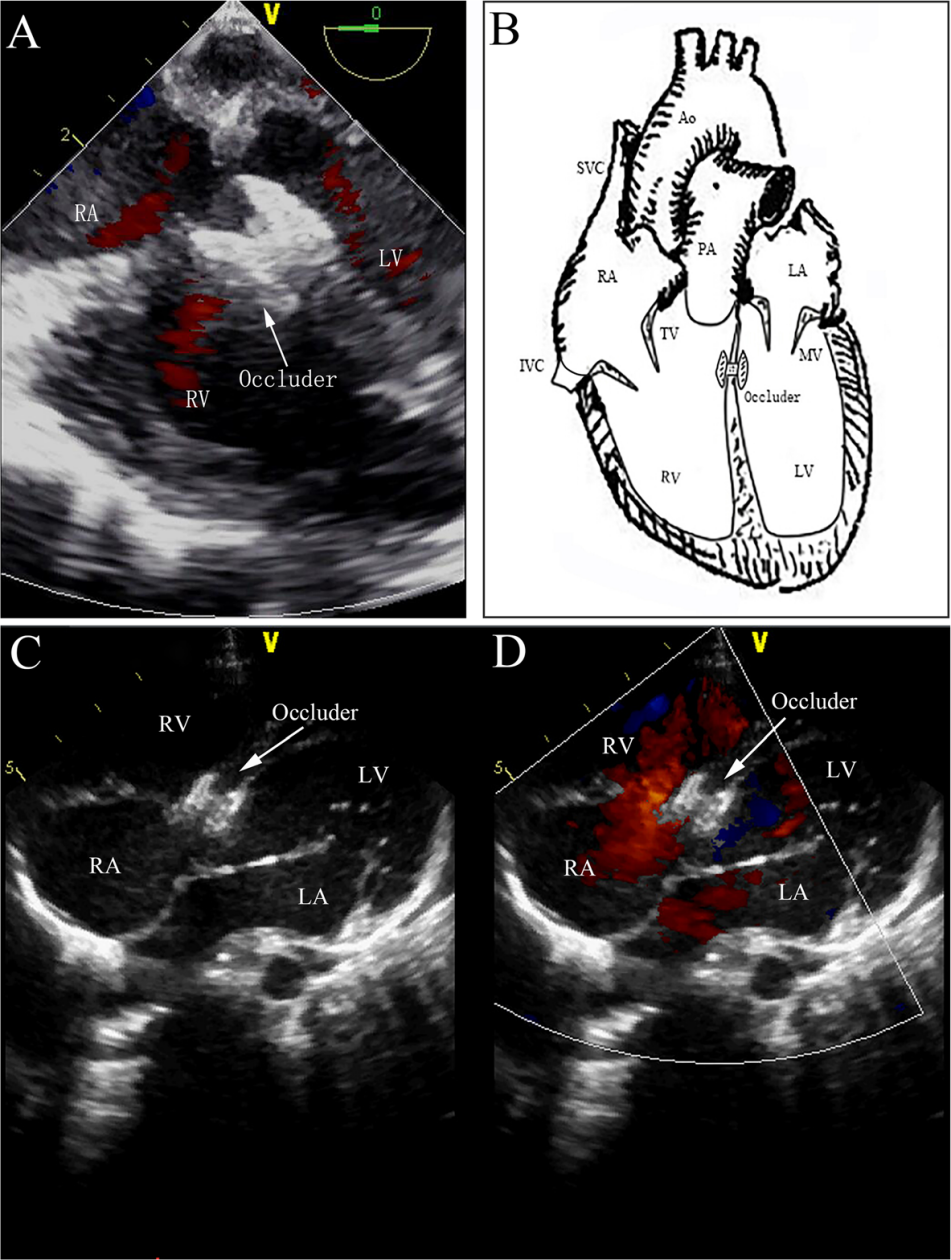
TEE (**a** and **b**) and TTE (**c** and **d**) were performed to ensure that there were no residual shunts or aortic regurgitation. LV: left ventricle; LA: left atrium; RA: right atrium; RV: right ventricle; AO: aorta; TV: tricuspid valve; MV: mitral valve; PA: pulmonary artery; SVC: superior vena cava; IVC: inferior vena cava

Femoral vein access was obtained using a 6-Fr sheath (Terumo Corporation, Tokyo, Japan). Heparinization was performed (125 U/kg) for anticoagulation. A 0.038­inch bent stiff guide wire (provided by Terumo Medical Corporation, Somerset, New Jersey, USA) was advanced from the inferior vena cava (IVC) via the right atrium (RA) into the superior vena cava (SVC) under TEE guidance using the bicaval view. A 5Fr JR 4 diagnostic catheter (provided by Cordis Corporation, Miami Lakes, Florida, USA) was subsequently advanced into RA over the wire. Then the guidewire was withdrawn progressively and the tip of catheter was tracked by the TEE short-axis view (Fig. 2A1 and A2). It may be necessary to adjust the direction of the catheter tip to advance through the tricuspid valve (TV) towards right ventricle (RV). The guidewire was then advanced into RV through the catheter (Fig. 2B1 and B2). The catheter was advanced into the RV over the guidewire, and the guidewire was then removed. Guided by TEE, along with the wire, the catheter’s direction was adjusted again to go through the outflow tract of RV towards main pulmonary artery (Fig. 2C1 and C2). The catheter was then gradually withdrawn and with the guidance of TEE, to make its tip point to the defect and the catheter advanced into the left ventricle (LV) (Fig. 2D1 and D2). The guidewire was guided slowly and gently through the VSD into the LV via the TEE four-chamber view (Fig. 2E1 and E2). We removed the vascular sheath and the right coronary catheter. The long delivery sheath (7F–8F) loaded with the dilator was advanced into the LV over the guidewire, and the dilator was then removed along with the guidewire, leaving just the tip of the sheath adjacent to the cardiac apex (Fig. 2F1 and F2). Thereafter, the occluder device was delivered along the sheath. Under TEE guidance, the sheath was then gently withdrawn to deploy the left disc in the LV (Fig. 2G1 and G2) and then pulled back until the disc was against the interventricular septum wall. The right side of the device was deployed by retracting the delivery sheath while applying slight tension on the cable (Fig. 2H1 and H2). Before the device was unscrewed, its correct and stable position was confirmed by TEE. Then TEE (Fig. 3a and b) and TTE (Fig. 3c and d) were performed to ensure that there were no residual shunts or aortic regurgitation. Bundle branch block was evaluated by electrocardiography during the procedure. If no postoperative complications (aortic regurgitation, complete atrioventricular block, or residual shunt) were found, both the loader sheath and the cable were withdrawn. The femoral vein was compressed for 5–8 min to stop the bleeding. A prophylactic antibiotic was administered during the procedure and on the day after. Moreover, aspirin (3 mg/kg/day) was routinely administered for 6 months.

If complications (aortic regurgitation, complete atrioventricular block, or residual shunt) were detected, the patients underwent open heart repair under CPB.

### Statistical analysis

Data are expressed as percentages for nominal variables and mean ± standard deviation for continuous variables. Statistical Program for Social Sciences (SPSS) for Windows, version 24.0 (IBM, Armonk, NY, USA), was used for statistical analysis.

## Results

### Procedural outcomes

All procedures were carried out in a standard theatre without fluoroscopy backup, but with a CPB unit stand­by. The transcatheter occlusion procedure under TEE guidance was successful in 44 (95.7%) patients (Additional file 1: Video S1). PmVSD occlusion failed because of a residual shunt (width > 2 mm; flow rate > 3 m/s) in 2 patients (VSD diameter, 4.5 mm and 4.6 mm; subaortic rim, 3 mm) with no improvement even after using a 6-mm, 7-mm, and 8-mm occluder, and then they underwent open heart surgery.


**Additional file 1: Video S1.** Procedure of percutaneous device closure of PmVSD. (AVI 58370 kb)


Symmetrical VSD occluder with a size (waist length) of 6 mm or 7 mm was used in this study. The mean procedural duration, post-operative mechanical ventilation duration, intensive care unit (ICU) residence, and in-hospital durations were 28.2 ± 8.7 min (range, 12.0–42.0 min), 65.2 ± 5.6 min (range, 56.0–78.0 min), 2.1 ± 0.1 h (mean, 2.0–2.4 h), and 2.7 ± 0.2 d (range, 2.5–3.0 d), respectively. There was no need for blood transfusion in any of our patients, who underwent percutaneous device closure (Table 1).

### Follow-up

The patients were followed up by clinical examination, echocardiography, and electrocardiography at 1, 3, and 6 months, and 1 and 2 years postoperatively. All patients survived without any peripheral vascular injury, acute complications, or severe adverse events (death, valve injury, complete atrioventricular block or embolism) during the early period or follow-up.

Complete atrioventricular blocks were not detected, whereas IRBBB was observed in 3 patients. A new IRBBB was discovered in 1 case during the first postoperative month, and the IRBBB resolved spontaneously and changed to normal rhythm in 3 patients during the third month of follow-up. All patients showed sinus rhythm in the period of 12–24 months follow-up.

Tricuspid regurgitation had decreased in 1 patient, whereas no new atrioventricular regurgitation was detected in the other patients at the first month of follow-up.

A trivial residual shunt (width, 1 mm; flow rate < 2.0 m/s) was detected in 1 patient on discharge. Fortunately, the residual shunt disappeared at the third month of follow-up (Table 1).

No other complications such as device dislocation, thrombosis, or obstruction of the left or right ventricular outflow tract were observed in the present study.

## Discussion

PmVSD is the most common subtype of VSD, and responsible for > 85% of such defects. Although surgical repair through median sternotomy on CPB has been regarded as the gold standard for treatment of pmVSDs, complications such as bleeding, wound infection and cerebrovascular accidents have been reported. Interest has grown in the development of new techniques that can replace traditional open-heart surgery for treatment of PmVSD. The well-accepted guidance for transcatheter VSD closure has been X-ray angiography. However, two major concerns remain with the conventional techniques. One is the high exposure to radiation, especially for infants and children [13, 14]. The other one is the increased potential risks of vascular complications caused by femoral artery puncturing for angiography [15, 16]. Arterial access in children is reported to cause a high rate of complication, of which a significant proportion requires intervention [17, 18]. Moreover, complications such as arterial disruption, or acute occlusion, may be even limb-threatening [19].

In an effort to reduce injury related to fluoroscopy and angiography, TEE has been used for guidance during perventricular occlusion of VSD in recent years. Intraoperative device closure of PmVSDs without radiation and CPB under guidance of TEE was first reported by Amin and colleagues [20], and has become an accepted modality of treatment in many cardiac centers [4, 21, 22]. However, this approach still presents certain disadvantages such as surgical incision, surgical complications, and scarring.

Despite separate solutions being proposed to either reduce radiation exposure and avoid arterial access, or reduce surgical incision and complications, there is no reported method that deals with these problems at the same time. Our study is the first to report that echocardiography can be used as the only guidance for transcatheter VSD closure without arterial access, radiation exposure, surgical incision or surgical scarring. This novel technique is used as an alternative to surgical repair of PmVSD in our cardiac center.

Compared with the traditional interventional methods, the advantages of this technique is sole used of the femoral vein approach, the lack of need to establish the arterial-venous wire loop and no risk of femoral artery related complications. Second, the device can be easily controlled and adjusted during the procedure. Percutaneous device closure under TEE guidance just needs to establish a shorter transfer track that involves the right femoral vein, IVC, RA, TVs, RV, VSD, and LV. Third, the inclusion of TEE imaging is easier and safer [23, 24]. And compared with TTE, the advantages of TEE are no obstruction of the chest wall and lung, clearer image, stronger color Doppler signal, more cardiac anatomy information, and no interference in the monitoring operation. TEE provides a clear view of vessels and routes during surgical procedures [25]. The vital part of percutaneous device closure is that the guidewire and delivery sheath should pass through the PmVSD under TEE imaging. TEE—performed immediately before, during, and after deployment of the occluder device—has been considered as a standard technique for this procedure [8]. The site, size, and rims of the defect; suitability of the device; and guidance of the guidewire and sheath through the PmVSD can be precisely assessed via TEE during the procedure. Furthermore, once the device is released, TEE can provide beneficial information for occlusion; a repeat image can also be obtained to assess the effectiveness of PmVSD closure, including the device position, the presence of a residual shunt, and aortic valve regurgitation. Any dislocation or residual shunting is easy to find and further adjustments can be made immediately. In addition, there is no risk of radiation exposure, particularly for children and surgeons, and advanced equipment is not needed, such as the machine of digital subtraction angiography to identify and track the route, and no risk of allergy and renal failure.

Four types of VSD occluder were designed. The symmetric occluder that is suitable for single-rupture PmVSD and not easy to cause atrioventricular block, is concentric, and both discs are 2 mm larger than the waist. The left disc of the small waist big edge-type occluder, which is suitable for multi-rupture PmVSD, is 4 mm larger than the right disc, which is 4 mm larger than the waist. The asymmetric occluder is suitable for subaortic VSD and has a platinum marker guiding device in the left disc for orientation. In order to avoid aortic valve injury, the left disc of the occluder is an ellipse with 0 mm towards the aorta, and the right disc is a circle with 2 mm larger than the waist. The muscular occluder is suitable for muscular VSD and its waist height is 7 mm rather than 3.5–4.5 mm.

The procedure time for the fluoroscopy­free transcatheter VSD closure ranged from 12 to 42 min. The difference of the operator’s performance view may account for this. Unlike the traditional procedure, the performance view in the fluoroscopy­free technique is TEE, which only provides two­dimensional (2D) views for operators. Although TEE has shown more advantages over fluoroscopy in the measurement of rims and dimensions of VSD, monitoring the deployment of the S-pmVSO and evaluation of the result of the implant, the biggest challenge for operators is to track the guidewire and sheath under the 2D view. Our initial experience confirms this. In our study, the device placement process was clearly visible using TEE for guidance, and well­timed placements can be precisely controlled by the operators. The main factors associated with procedure time include:1) the process to reach the defects, because there may be some difficulty to track the tip of the catheter and/ or guidewire with TEE; 2) the course of device deployment, as the angle between the sheath and ventricular septum may not be good enough, so that redeployment may be needed. Although a longer operating time was needed in certain cases, especially easy in our experience, the procedure time decreased remarkably as operators gained experience. The learning curve is very short for operators.

Residual shunt is the most frequent complication of percutaneous transcatheter closure of PmVSD. Proper selection of patient and device is mandatory for prevention of this inherent complication. In our study, all procedures were carried out in a standard theatre with a CPB unit stand­by. Thus, a patient could be converted to open heart surgery immediately in order to retrieve the device and repair the PmVSD.

Complete atrioventricular block is a significant problem for occlusion, particularly when using the Amplatzer occluder [26]. No cases of complete atrioventricular block were detected in the present study. We believe that this observation was related to the procedure within the RV, which stimulated the right bundle branch block around the VSD. Care must be taken not to damage the tricuspid valve/chordae. In order to do so, clockwise torque on the catheter is occasionally required to obtain the right direction while advancing the tip of the catheter towards the right ventricle. If resistance is noted, the catheter should be turned clockwise and adjusted to avoid valve injury.

One possible disadvantage of this new technique is that the procedure is performed under general anaesthesia. However, we consider this only a minor drawback because general anaesthesia might also be needed for young children when performing traditional transcatheter closure or surgical repair of PmVSDs. Moreover, the data were from a single-centered study, the outcome and the superiority of this new strategy should be validated by prospective multi-centered randomized controlled trials.

## Conclusions

Percutaneous device closure of PmVSDs under TEE guidance solely by femoral vein approach is effective and safe, avoids radiation exposure, potential arterial complications and a surgical incision.

## Data Availability

The datasets used and/or analysed during the current study are available from the corresponding author on reasonable request.

## Abbreviations

CPB: Cardiopulmonary bypass
ICU: Intensive care unit
IRBBB: Incomplete right bundle branch block
LV: Left ventricle
PmVSDs: Perimembranous ventricular septal defects
RV: Right ventricle
RVOT: Right ventricular outflow tract
SPSS: Statistical Program for Social Sciences
SVC: Superior vena cava
TEE: Transesophageal echocardiography
TV: Tricuspid valve
VSD: Ventricular septal defect
